# Microbial *hara-kiri*: Exploiting lysosomal cell death in malaria parasites

**DOI:** 10.15698/mic2015.02.186

**Published:** 2015-01-12

**Authors:** Jun-Hong Ch’ng, Johan Ursing, Kevin Shyong-Wei Tan

**Affiliations:** 1 Department of Microbiology, National University of Singapore, Singapore.; 2 Department of Microbiology, Tumor and Cell Biology, Karolinska Institute, Stockholm, Sweden.

**Keywords:** malaria, Plasmodium falciparum, chloroquine, lysosomal cell death, programmed cell death, digestive vacuole, repositioning

## Abstract

The antimalarial drug chloroquine (CQ) has been sidelined in the fight against *falciparum *malaria due to wide-spread CQ resistance. Replacement drugs like sulfadoxine, pyrimethamine and mefloquine have also since been surpassed with the evolution of multi-drug resistant parasites. Even the currently recommended artemisinin-based combination therapies show signs of compromise due to the recent spread of artemisinin delayed-clearance parasites. Though there have been promising breakthroughs in the pursuit of new effective antimalarials, the development and strategic deployment of such novel chemical entities takes time. We therefore argue that there is a crucial need to re-examine the usefulness of ‘outdated’ drugs like chloroquine, and explore if they might be effective alternative therapies in the interim. We suggest that a novel parasite cell death (pCD) pathway may be exploited through the reformulation of CQ to address this need.

As an amphiphilic weak base, chloroquine (CQ) accumulates preferentially in the acidic lysosome-like compartment of the parasite called the digestive vacuole (DV). Therein, CQ inhibits the polymerization of heme (a by-product of the parasite’s digestion of hemoglobin) into inert hemozoin. Without this neutralizing reaction, cytotoxic heme accumulates within the parasite and kills it. In CQ-resistant parasites, mutations to the chloroquine resistance transporter (PfCRT) allow protonated CQ to be pumped out of the DV, permitting hemozoin polymerization to continue and the parasites to survive. Such mutations increase the concentration of drug required to kill the parasite (IC_50_ increases from tens of nanomolar to hundreds of nanomolar) thereby decreasing the efficacy of the recommended dosing regimen.

In our previous studies, we suggested an alternative antimalarial mechanism of CQ that is only observed at concentrations exceeding nanomolar levels and does not rely on the build-up of heme to kill parasites. At such micromolar levels, CQ overcomes the saturable PfCRT and accumulates within the DV to very high concentrations resulting in permeabilization of the DV membrane. There is a subsequent release of DV cysteine proteases into the parasite cytoplasm which leads to mitochondrial depolarization, DNA cleavage and cell death. This parasite cell death (pCD) pathway shares many similarities with mammalian lysosomal cell death and can also be induced by other lysosomotropic compounds that do not affect hemozoin polymerization or have any known parasite targets.

In our recent publication, we first demonstrated that this novel pCD pathway was also effective in CQ-resistant (and multi-drug resistant) parasites by validating the findings in multiple lab and field parasites. Secondly, using drug-pulse assays where trophozoite-infected cells were treated with a high concentration of CQ (above 1 μM) for 4 hrs, we showed that the pCD pathway was rapidly induced. Given that blood CQ levels reach micromolar levels in the initial hours after dosing, it is possible that CQ-induced pCD is the primary initial anti-parasitic mechanism of CQ action. This hypothesis could be confirmed by looking for pCD features in parasites collected from infected patients being treated with CQ while monitoring plasma CQ levels. This is difficult to do since there are few places in the world that continue to use CQ in the treatment of *falciparum *malaria and we looked to demonstrate the utility of the pathway in murine malaria models instead.

To establish murine malaria models, we used parasites harvested from *P. yoelii- *and *P. berghei*-infected mice and found that permeabilization of the DV occurs after 4 hrs exposure at micromolar CQ levels in both parasite species *ex vivo*, much like in *P. falciparum*. After this drug pulse, parasites that had been exposed to micromolar (but not nanomolar) levels of CQ showed markedly reduced reinvasion rates when injected into naive mice, agreeing with our hypothesis that parasite DV permeabilization leads to loss of cell viability.

To simulate what would happen in a CQ-treated patient, we fed *P. berghei*- and *P. yoelii*-infected mice with varying doses of CQ (in a single bolus), harvested the parasites and assayed them for DV permeabilization. At 50 mg/kg (five times the standard first dose), murine parasites showed an increased proportion of DV permeabilization. Corresponding plasma CQ concentrations for these mice was assayed and estimated to exceed 1 μM during the first four hours. This suggests that micromolar CQ levels triggered DV permeabilization *in vivo*, as was the case *in vitro*. Moreover, a rapid reduction in parasite reinvasion was also evident in *P. berghei*-infected mice as early as 6 hrs post-treatment.

Although we have demonstrated the possibility of using an alternative antimalarial mechanism of CQ by elevating blood levels of the drug in two murine malaria models, any attempt to escalate patient dosage needs to be carefully considered. Given the variability of CQ pharmacokinetics, low therapeutic index of CQ and the risk of cardiac dysfunction, increasing the bolus dose could lead to serious adverse side effects. Moreover, it has been previously demonstrated that increasing CQ doses does not necessarily restore the efficacy of CQ, with high rates of recrudescence reported in several trials. These concerns highlight the value of looking for novel lysosomotropic compounds which may be able to specifically disrupt the parasite DV without endangering patients - an effort which may uncover an entirely new class of antimalarials. But more so, these concerns beckon us to consider the importance of drug pharmacokinetics in relation to pharmacodynamics.

To effectively use CQ in the treatment of drug-resistant malaria, we believe that it will be necessary to both increase the blood concentration of the drug and sustain these levels at micromolar levels for several consecutive days. The increased CQ concentration would result in parasites dying by pCD (in addition to heme cytotoxicity) while the prolonged exposure would mean that early-stage parasites (that do not have mature DVs) will not be able to circumvent this mechanism by waiting for CQ levels to be depleted. We suggest that sustainable high CQ blood levels may be achieved by using multiple smaller dosing or by designing a slow-release formulation of CQ. Such approaches may reduce the initial spike in blood CQ levels (which can cause severe adverse effects) while maintaining a prolonged micromolar quantity of the drug to induce parasite cell death.

Coincidently, this strategy was utilized in Guinea-Bissau up to 2008 when approximately three times the standard total CQ dose was routinely given as 2-3 smaller daily doses over five days. This dosing was well tolerated and commonly resulted in micromolar day 7 CQ concentrations that probably induced pCD *in vivo*. Interestingly, the prevalence of *P. falciparum *resistant to standard dose CQ remained unusually low (25%) in Guinea-Bissau, suggesting that the routine use of higher CQ doses hindered the spread of *P. falciparum *resistance genes and did not lead to the development of highly resistant *P. falciparum*.

The clinical data from Guinea-Bissau is in line with experimental data and suggests that micromolar CQ concentrations can be effective for the treatment of drug resistant *P. falciparum*, quite possibly through the dual antimalarial mechanisms of pCD and inhibition of hemozoin polymerization. Though re-formulation of CQ to exploit the pCD pathway will require further clinical validation, the prospect of using CQ (a well characterized, tolerated, stable and affordable drug) to manage multi-drug resistant malaria warrants further consideration.

